# Genome-wide dynamic network analysis reveals a critical transition state of flower development in *Arabidopsis*

**DOI:** 10.1186/s12870-018-1589-6

**Published:** 2019-01-07

**Authors:** Fuping Zhang, Xiaoping Liu, Aidi Zhang, Zhonglin Jiang, Luonan Chen, Xiujun Zhang

**Affiliations:** 1 Key Laboratory of Plant Germplasm Enhancement and Specially Agriculture, Wuhan Botanical Garden, Chinese Academy of Sciences, Wuhan, 430074 China; 2 University of Chinese Academy of Sciences, Beijing, 10049 China; 3 Key Laboratory of Systems Biology, Innovation Center for Cell Signaling Network, Institute of Biochemistry and Cell Biology, Shanghai Institutes for Biological Sciences, Chinese Academy of Sciences, Shanghai, 200031 China

**Keywords:** Time-course gene expression data, Gene regulatory network (GRN), Dynamical network biomarker (DNB), Phase transition, Flower development

## Abstract

**Background:**

The flowering transition which is controlled by a complex and intricate gene regulatory network plays an important role in the reproduction for offspring of plants. It is a challenge to identify the critical transition state as well as the genes that control the transition of flower development. With the emergence of massively parallel sequencing, a great number of time-course transcriptome data greatly facilitate the exploration of the developmental phase transition in plants. Although some network-based bioinformatics analyses attempted to identify the genes that control the phase transition, they generally overlooked the dynamics of regulation and resulted in unreliable results. In addition, the results of these methods cannot be self-explained.

**Results:**

In this work, to reveal a critical transition state and identify the transition-specific genes of flower development, we implemented a genome-wide dynamic network analysis on temporal gene expression data in *Arabidopsis* by dynamic network biomarker (DNB) method. In the analysis, DNB model which can exploit collective fluctuations and correlations of different metabolites at a network level was used to detect the imminent critical transition state or the tipping point. The genes that control the phase transition can be identified by the difference of weighted correlations between the genes interested and the other genes in the global network. To construct the gene regulatory network controlling the flowering transition, we applied NARROMI algorithm which can reduce the noisy, redundant and indirect regulations on the expression data of the transition-specific genes. In the results, the critical transition state detected during the formation of flowers corresponded to the development of flowering on the 7th to 9th day in *Arabidopsis*. Among of 233 genes identified to be highly fluctuated at the transition state, a high percentage of genes with maximum expression in pollen was detected, and 24 genes were validated to participate in stress reaction process, as well as other floral-related pathways. Composed of three major subnetworks, a gene regulatory network with 150 nodes and 225 edges was found to be highly correlated with flowering transition. The gene ontology (GO) annotation of pathway enrichment analysis revealed that the identified genes are enriched in the catalytic activity, metabolic process and cellular process.

**Conclusions:**

This study provides a novel insight to identify the real causality of the phase transition with genome-wide dynamic network analysis.

**Electronic supplementary material:**

The online version of this article (10.1186/s12870-018-1589-6) contains supplementary material, which is available to authorized users.

## Background

The flowering plants undergo a succession of developmental phases during their life cycle including germination, seedling, flowering and fruiting. As a special pattern of plant development, phase transition is crucial for the survival and reproduction of the plant, and the failure implementation of phase transition will result in the dysfunction of development [1–3]. The phase transitions related to plant development include the seed-to-seedling transition [4], the juvenile-to-adult vegetative transition [5], the vegetative-to-reproductive transition [6], the heterotrophic-to-autotrophic transition [7], the initiation-to-maturation floral transition [8], and so on. These developmental phase transitions form the main functional mechanisms in plants [9].

Among the developmental phase transitions, flowering transition has been extensively studied by the experimental biologists because flowering is an irreversible transformation from vegetative to reproductive growth, an important qualitative process, a key stage of plant development, and a reproduction for offspring [10]. The flowering process include three phases, i.e. the floral induction phase, the floral primordia phase, and the floral organs development phase [8]. During these phases, a series of genes such as flowering time controlling genes, meristem identity genes and floral organ identity genes are involved in the regulation of flowering transition [11].

To understand the mechanism of flowering transition, a series of intensive studies on flower development were implemented in recent years and the results provided some crucial clues [12–15]. For example, it is reported that flower development is firstly characterized by dramatic changes in morphology such as floral patterning, floral organ size and floral organ specification. These changes are regulated by a great number of regulators like transcriptional factors and miRNAs [8, 16]. The growth cone is transformed from differentiation of leaves to differentiation of flower buds in the ontogeny of higher plant, which marks the transition from vegetative growth to reproductive growth or the beginning of flower development [11].

In recent years, it has become increasingly clear that the phase transitions were controlled by distinct genetic circuit incorporating endogenous and environmental cues, such as the interaction between NF-YC and CLF, the interaction between miR156 and miR173 [9], and so on. These genes are involved the regulation of phase transition by undergoing regular changes to form a complex gene regulatory network (GRN) [11]. The flowering mechanism of the model plant *Arabidopsis* is relatively clear with a flowering gene regulatory network involving signal transduction networks such as photoperiod, autonomous, vernalization, and gibberellin pathways [17]. More and more researches demonstrate that the flowering developmental gene regulatory networks provide an important breakthrough for better understanding the inherent mechanism of flowering transition.

Attribute to the advances of next-generational sequencing technology, bioinformatics and computational biology expedited the process of biological research by genome-wide transcriptome analysis [18]. Transcriptome analysis provides a perfect approach to study biological problem because it gives not only a global view of gene expression patterns including biological function enrichment but also a predictive dimension by identifying a set of co-expressed genes [19, 20]. With above advantages, bioinformatics analysis of high-throughput transcriptome data provides a powerful tool to address the issues in exploring the mechanism of the phase transition in a genome-wide scale [21].

The transcriptome analysis by predicting GRN has been used to discover the regulatory mechanism in plants [22]. Different types of tools have been developed to infer large-scale GRNs from the gene expression data, such as correlation-based methods [23], mutual information-based method [24, 25] and regression-based methods [26–28]. Our group previously have developed some GRN inference tools such as NARROMI [29], PCA-CMI [30] and CMI2NI [31, 32]. These methods have greatly improved the accuracy of GRN inference by reducing the noisy, redundant and indirect regulations.

Recently, the predictive GRN was used to analyze the phase transition and some novel genes controlling the seed-to-seedling phase transition in *Arabidopsis* were identified [19, 33]. Not only that, the *Arabidopsis* floral transition process was deciphered by integrating a protein-protein interaction network and gene expression data [34]. For the traditional analysis methods, it is difficult to catch up the truth of complex phase transitions with the traits of temporal and spatial dynamics with polygene interactions. Moreover, the research has focused on the mechanism of flower development under undifferentiated stem cells but not the mechanism of floral organ transformation to maturation [35]. In addition, the current methods have not fully used the dynamics character of phase transition and not necessarily catch the real regulators controlling the phase transitions [36]. While the time-course transcriptome data with multiple replicates provide the materials for the bioinformatics analysis to identify the regulators of phase transition [37, 38].

With more and more time-course high-throughput transcriptome data available, some dynamic network analysis-based bioinformatics tools have been developed and widely used to study the complex biological mechanism [39, 40]. For example, the dynamic network biomarker identification tool, named DNB, was developed to detect the biomarker [41]. In this method, a complex biological process (e.g., differentiation processes, aging processes, and phase transition of the cell cycle, etc.) is divided into three phases or states, i.e. the before-transition state, the critical transition state, and the after-transition state [42]. In a focal system, a drastic or qualitative transition from the critical transition state to the post-transition state corresponds to a bifurcation point in the dynamical systems theory [43]. When the system is near to a critical point, a dominant group of biomarkers become bifurcate. The biomarkers can be defined by three conditions: isolated subnetwork or functional module, high fluctuation of the members, strong correlation between any pair of its members while weak correlation between members and non-members [44]. Dynamic network analysis with a solid theory support has been successfully applied to real biological data [43–45].

In this work, to provide a new insight into the flowering developmental transition, we performed a genome-wide dynamic network analysis on the time-course gene expression data in *Arabidopsis*. The dataset with 14 stages of flowering from initiation to maturity and 3 replicates for each stage provides a dynamic process of flowering development. The genes that control the phase transition can be identified by the difference of weighted correlations between the genes interested and the other genes in the global network. To construct the gene regulatory network controlling the flowering transition, we applied NARROMI algorithm which can reduce the noisy, redundant and indirect regulations on the expression data of the transition-specific genes. The results were validated by biological experimental analysis and the predicted transition state is consistent with the real transition state of the flowering in phenotype. We also performed a similar analysis of temporal gene expression profiling dataset of rice flower development to support the conclusions drawn. Ultimately, we detected a critical transition state of flower development in rice. This work provides a novel insight into the identification of the transition state and the key causal genes that control flowering transition by dynamic network analysis of time-course gene expression data.

## Results

### A dynamic network biomarker(DNB) model for detecting critical transition state of complex biological systems

**Fig. 1:**
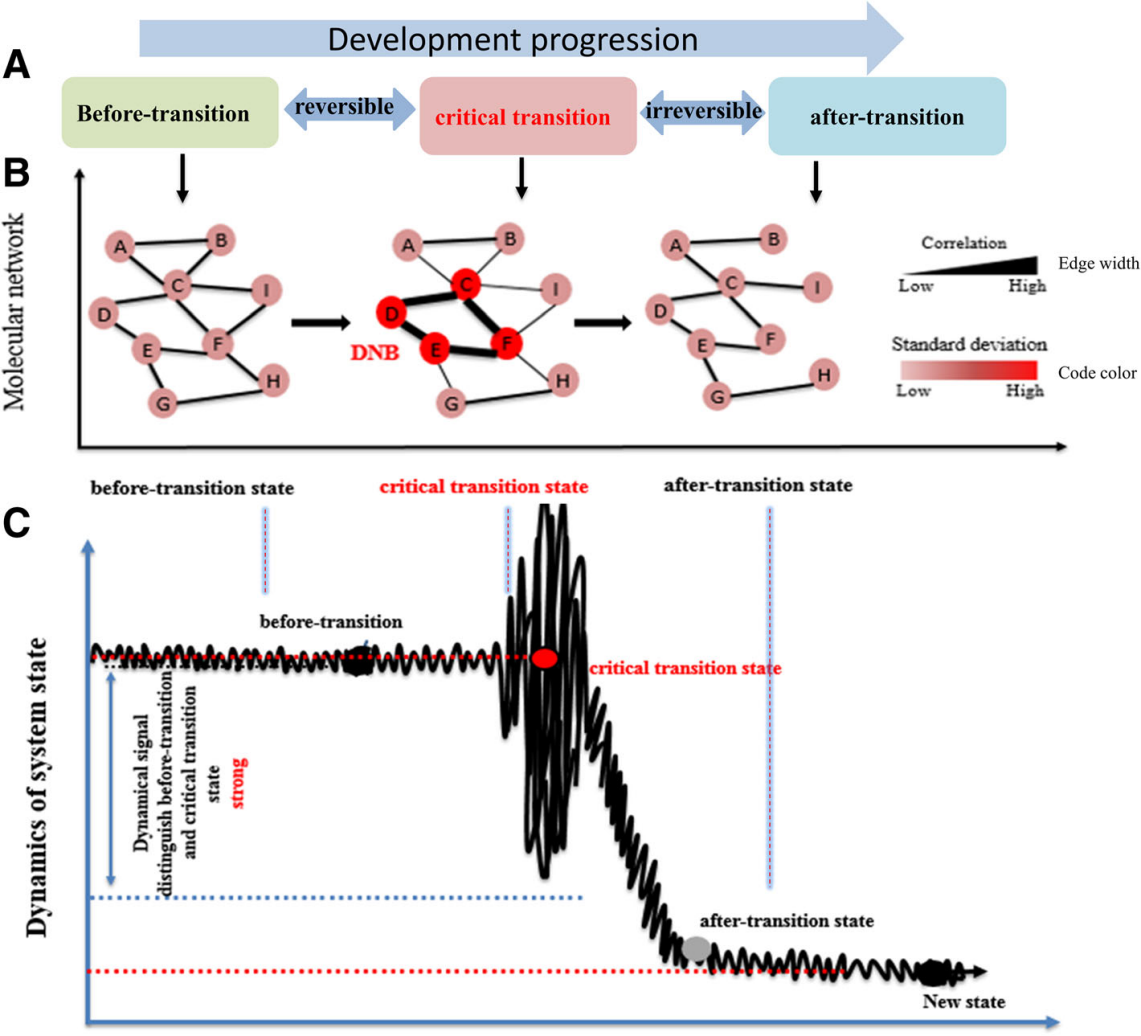
The outline representation of critical transition state by DNB based on time-series gene expression data. **a** The progression of *Arabidopsis* flower development from the time of initiation to maturation can be divided into three states, i.e., the before-transition state, the critical transition state, and the after-transition state. A system at the before-transition state or the after-transition state is stable with high resilience. **b** In the critical transition state, deviations of the DNB members increase drastically, and the correlation between any two molecules increases. This critical phenomenon does not appear at the before-transition and the after-transition states. **c** The DNB members show large fluctuations in their expressions at the critical transition state, compared with smaller fluctuations of the expressions at the before- or after- transition states

In the phase transition model, a complex biological process (e.g., differentiation processes, aging processes, and changes in the phases of the cell cycle) can be generally divided into the three stages, i.e. the before-transition state, the critical transition state, and the after-transition state [42]. The before-transition state is relatively stable but may change gradually because of certain internal and external motivators (Fig. 1). The critical transition state can be understood as the limit of the before-transition state just before a critical transition. Not only that, but it can be easily reverted to the before-transition state by the appropriate external interventions and thus it is considered reversible. While the after-transition state is stable and irreversible even with intensive interventions. Through the identification of dynamic network biomarkers(DNBs), we can control the complex system to avoid the development of the system toward the bad state. In other words, controlling the “causes” of the development of the system can avoid the occurrence of “effects” or change the shape of “effects”. It is crucial to crush the source of trouble in the egg to achieve the ideal effect of achieving effective control of complex systems at a lower cost.

The theoretical basis for detecting DNBs was summarized by the following conditions (Fig. 1b and c). Briefly, DNB is an observable subnetwork composed of a set of special molecules in the original system which satisfies the following four requirements only at the critical state [46]:

1. DNB molecules or markers are highly fluctuating, i.e. deviations of the DNB members(genes) increase drastically (high SD_*d*_).
2. All members of DNBs are highly co-expressed, i.e. correlations among DNB members become stronger (high PCC_*d*_).
3. DNB members are almost independent of non-DNB members. DNB is an isolated subnetwork or functional module, i.e. the correlation between any DNB molecules and other non-DNB decreases (low PCC_*o*_).
4. There are no drastic deviations or correlations among all non-DNB molecules in the system.

To detect a reliable and unambiguous signal of the critical transition state, a composite index(CI) is proposed as follows

$$ \mathrm{CI}=\left({\mathrm{SD}}_d\cdotp {\mathrm{PCC}}_d\right)/{\mathrm{PCC}}_o, $$

where SD_*d*_ and PCC_*d*_ are average standard deviation(SD) and average *Pearson* correlation coefficient(PCC) of all molecules in DNB module *d* respectively, while PCC_*o*_ is the average correlation between molecules in *d* and others that are not in *d*. When a biological system approaches a critical transition state, CI provides a reliable and significant early-warning signal. Among all the responsive CI modules, the maximum one is most likely to be the DNB that corresponds to the critical stage of the system.

### Pipeline of genome-wide dynamic network analysis

To provide a new insight into the flowering developmental transition, we performed a genome-wide dynamic network analysis on the time-course gene expression data in *Arabidopsis*. The dataset with 14 stages of flowering from initiation to maturity and 3 replicates for each stage provides a dynamic process of flowering development. However, the dynamic network analysis to effectively reveal the critical developmental stage with a key sub-network is still a challenge [8]. In addition, the data with matched case and control samples are not available. Therefore, the effective data processing become crucial to identify the DNBs for the flower developmental transition.

**Fig. 2:**
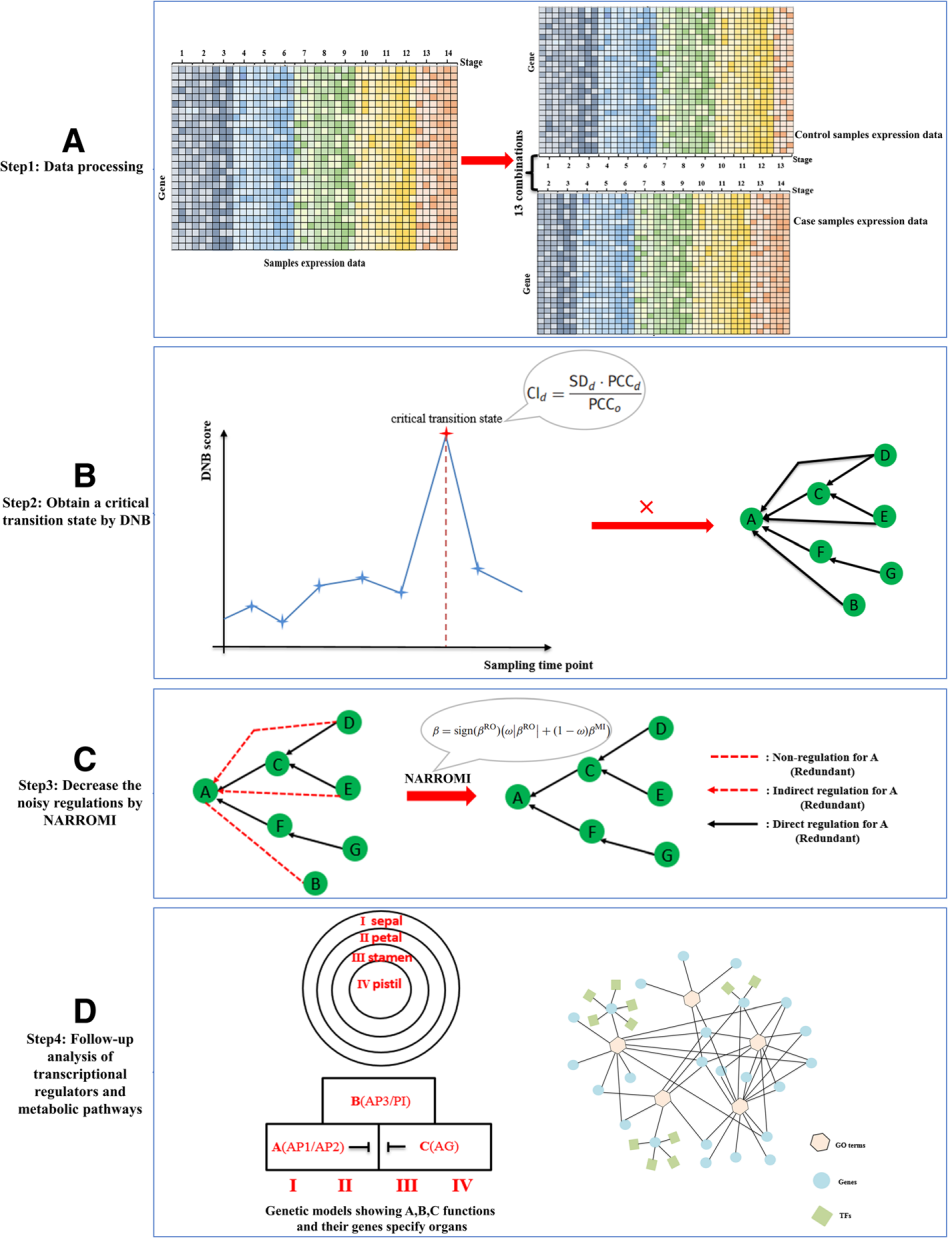
The flow diagram for revealing the critical transition state. **a** With samples in previous and the current time points designated as the control and case sample respectively. The 14 different developmental stages from initial to mature were divided into 13 combinations. **b** We applied DNB method to reveal a critical differentiation state of *Arabidopsis* flower development by comparing the control and case samples. **c** To construct the gene regulatory network controlling the flowering transition, we applied NARROMI algorithm on the expression data of the transition-specific genes. The NARROMI algorithm removes noisy regulations with low pair-wise correlations and redundant regulations from indirect regulators using ordinary differential equation-based recursive optimization (RO) and information theory-based mutual information (MI), respectively. The dotted line without arrowhead denotes non-regulation (redundant), the dotted arrow denotes the indirect regulation (redundant) and the solid arrow denotes the true regulation. **d** We also analyzed key regulatory factors and key metabolic pathways which were closely related to the phase transition of *Arabidopsis* flowering development from the time of initiation to maturation time

Previous studies have shown that *Arabidopsis* flowers are sequentially activated so that the flowers in their inflorescence are at different stages of development [47]. It also has been found that the top-head flowers of the *Arabidopsis* inflorescence remain quite synchronized throughout the flower development and therefore the time-series gene expression data of flowering could be obtained [8]. To overcome the lack of data with the matched case and control samples, the sample in former time point was designated as the control sample, and the neighbor later sample was designed as case sample. And then the 14 different developmental stages from initial to mature were divided into 13 case-control combinations with which we can detect the phase transition of Arabidopsis flower development (Fig. 2a).

The DNB model which is powerful than the traditional differential expression analysis was applied to detect the critical differentiation state of flower development from the time of initiation to maturation in *Arabidopsis* (Fig. 2b). NARROMI algorithm which can reduce the noisy, redundant and indirect regulations on the expression data of the transition-specific genes was used to construct the gene regulatory network controlling the flowering transition (Fig. 2c) [29]. With the identified DNBs, key regulatory factors and metabolic pathways closely related to the phase transition of *Arabidopsis* flower development from the time of initiation to maturation were analyzed (Fig. 2d).

### The identified critical transition state in *Arabidopsis* flower development

Identifying the critical differentiation stage or critical transition state of *Arabidopsis* flower development from the time of initiation to maturation is crucial to clarify the molecular mechanism that regulates plant flowering. However, the traditional methods based on differential expression analysis failed to detect the critical state due to the lack of significant differential expressions of molecules in the before-transition and critical transition states. To overcome this problem, the DNB model was developed to measure collective fluctuations of molecules taking place of the traditional differential expression analysis (see Methods). Traditional methods often rely on the differential expressions of molecules, while the DNB model uses both differential correlations and differential deviations among molecules [41, 48]. Despite of the weak differential expression among genes in the before-transition state and the critical transition state, significant differential correlations and deviations among genes existed in these two states.

**Fig. 3:**
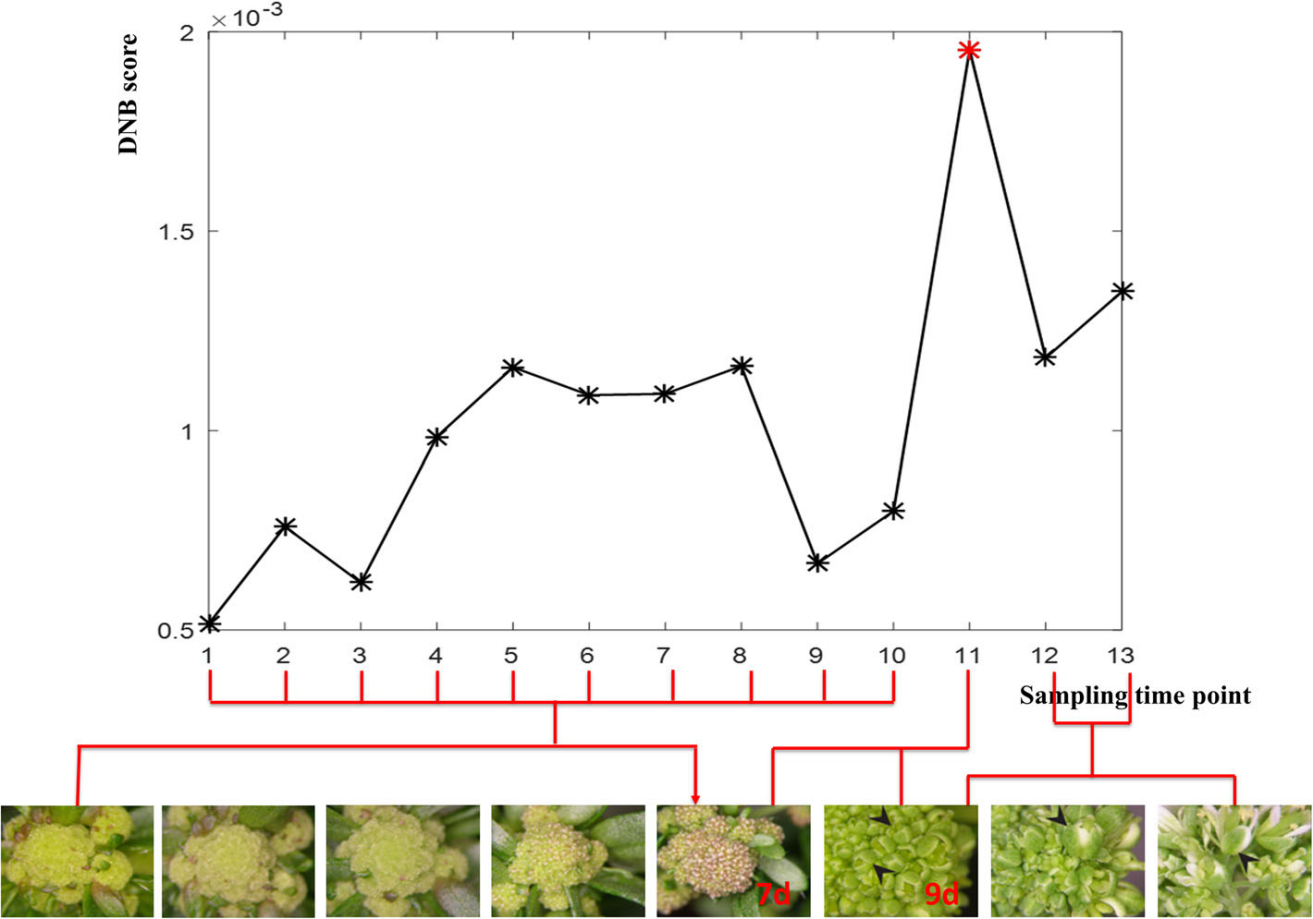
Identification of the critical transition state of flower development in *Arabidopsis thaliana*. The DNB scores at 13 sampling time points is shown in the FIGURE. For the black curve, the DNB score increased sharply from the 10th point and reached the peak at the 11th point. The 11th sampling time point annotated by the red star is designated as a critical transition state, which corresponds to the comparison of growth on the 7th to 9th day of Arabidopsis flower development

We implemented the DNB model on the *Arabidopsis* flower development datasets (NCBI access no. GSE64581) to identify the critical transition and the genes of flower development from the time of initiation to maturation. Specifically, we compared the samples in previous time point as the control sample with the samples in current time point as the case sample. Ultimately, the 14 different developmental stages from initial to mature were divide into 13 sampling time points. The critical transition state of *Arabidopsis* flowering was detected in the before-transition and after-transition state at the 11th sampling point of the flower formation shown by the red star (Fig. 3).

The 11th sampling time point shown in Fig. 3 corresponded to the comparison of growth on the 7th to 9th day of *Arabidopsis* flower development. Moreover, the development of flower inflorescence was largely synchronous until day 7. For the later time-points, only the development of flowers at the tip of the inflorescence (arrowheads) remained synchronized after phenotypic assessment (Fig. 3) [8]. In order to verify the biological and statistical significance of the identified DNBs, we conducted bootstrap analysis and the results showed that the identified DNBs for flowering development in *Arabidopsis* were highly significant compared with the randomly chosen gene sets (Additional file 1: Figure S4).

### The identified genes controlling the transition of *Arabidopsis* flower development

Ryan et al. revealed that the development of Arabidopsis flower inflorescence was largely synchronous until day 7. After that, only the development of flowers at the tip of the inflorescence remained synchronized after phenotypic assessment [8]. As result of our analysis, the predicted critical transition state from timepoint 7th to 9th day was consistent with their experimental results. In other words, the critical transition state detected during the formation of flowers corresponded to the development of flowering in *Arabidopsis*. All identified DNB members corresponding to the critical state of flower formation were listed in Additional file 2: Figure S1, and the detailed description of DNB members was listed in Additional file 3: Figure S2. Functional categories for up- and down-regulated genes in DNB module were shown in Table 1, these genes were crucial for flowering transition. Moreover, functional categories for other genes in DNB module were shown in Additional file 4: Table S1.

**Table 1:** Functional categories for up- and down-regulated genes in the DNB module of *Arabidopsis* flower development

Gene name	Fold change	Transition state	Description
Transcription factors			
RAP2.6	0.73	down-regulated	AP2/B3-like, related to AP2 6(RAP2.6)
AGL46	0.19	down-regulated	MADS box transcription factor
AT2G31370	1.74	up-regulated	Basic-leucine zipper transcription factor
AT5G46030	2.12	up-regulated	transcription elongation factor-like protein
AT4G33280	0.71	down-regulated	AP2/B3-like transcriptional factor family protein
AT1G21580	1.66	up-regulated	Zinc finger C-×8-C-×5-C-×3-H type family
ZAP1	4.05	up-regulated	zinc-dependent activator protein-1
WRKY36	0.07	down-regulated	WRKY DNA-binding protein
AT1G59675	1.21	up-regulated	F-box family protein
NAP	2.37	up-regulated	NAC-like, activated by AP3/PI(NAP)
AT1G76590	1.63	up-regulated	PLATZ transcription factor family protein
RIE1	0.28	down-regulated	RING-finger protein for embryogenesis
AT4G25610	0.35	down-regulated	C2H2-like zinc finger protein
Protein kinase activity			
AT3G46270	3.83	up-regulated	receptor protein kinase-like protein
AT3G15240	3.61	up-regulated	Serine/threonine-protein kinase WNK
			(With No Lysine)-like protein
AT1G67720	0.42	down-regulated	Leucine-rich repeat protein kinase family protein
AT3G23310	0.75	down-regulated	AGC kinase family protein
Ras signaling pathway			
AGL46	0.19	down-regulated	MADS box transcription factor
Plant hormone signal transduction			
SHY2	0.61	down-regulated	AUX/IAA transcriptional regulator family protein
AIR12	0.79	down-regulated	auxin-induced in root cultures-like protein
IAA18	1.98	up-regulated	indole-3-acetic acid inducible 18
ABA signaling pathway			
HIS1–3	0.79	down-regulated	histone H1–3
SLAH2	1.22	up-regulated	SLAC1 homologue 2
AT4G25610	0.35	down-regulated	C2H2-like zinc finger protein
AT4G33280	0.71	down-regulated	AP2/B3-like transcriptional factor family protein
AT1G59675	1.21	up-regulated	F-box family protein

### A more reliable gene co-expression network controlling critical transition

A DNB module containing 233 genes about *Arabidopsis* flower development from the time of initiation to maturation was detected using the DNB model [41]. With the gene expression data of these transition-specific genes, we used NARROMI algorithm which can remove the redundant and indirect regulations to construct the gene co-expression network controlling the flowering transition. Network inference file was listed in Additional file 5: Table S3, and we got two new files by processing the file. The degree of correlation between each pair of DNB members of Arabidopsis flower development was listed in Additional file 6: Table S4, and the node properties file for the network was listed in Additional file 7: Table S5.

**Fig. 4:**
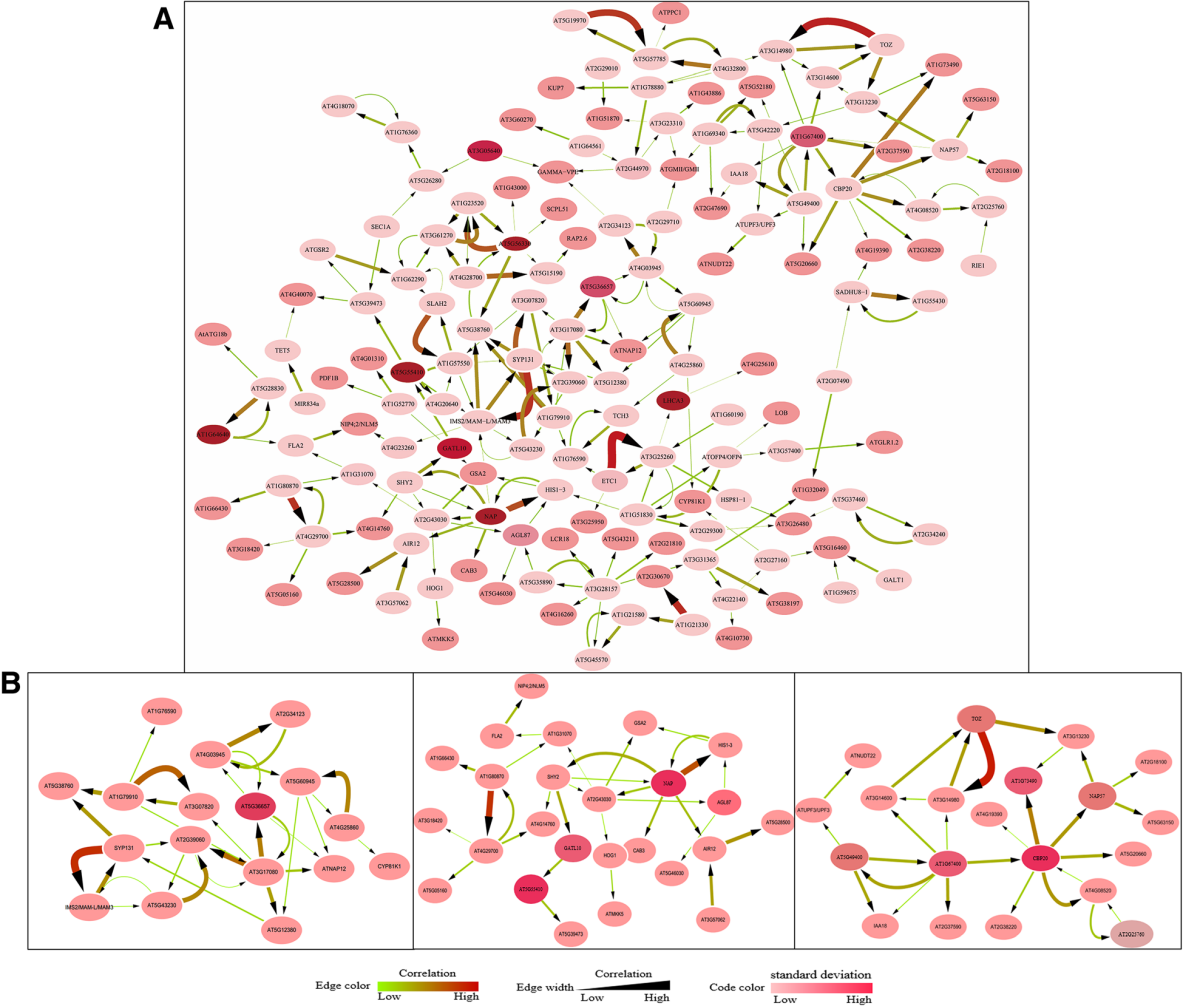
DNB members and correlations among these molecules were visualized in molecular networks by using Cytoscape. Node color reflects the standard deviation of the corresponding genes. The strength of correlations is represented by edge width, where a wider edge corresponds to a higher correlation. For clarity, the strength of correlations was also reflected by edge color. **a** A more reliable co-expression gene regulatory network of the Arabidopsis floral transition. **b** Three key subnetworks in DNB given separately

By applying the NARROMI approach to the 233 genes of the DNB module, we constructed a more accurate and reliable gene regulatory network of the *Arabidopsis* flowering transition. The file of gene regulatory network was listed in Additional file 8: Table S6. Ultimately, the members of DNB module and the correlations among members were visualized in a molecular network consisting of 150 nodes with 225 edges. In the network, node color reflects the standard deviation of the corresponding genes and the strength of correlations reflected by edge width, where a wider edge corresponds to a higher correlation (Fig. 4a). Three key subnetworks in the DNB module are clustered separately because the standard deviation of the corresponding genes and the strength of correlations in these subnetworks were significantly different, and we deemed these distinct regions as the most representative of this critical transition (Fig. 4b).

### Functional classification of DNB members during the critical transition state

The critical transition state during the formation of flowers was identified by DNB approach, and it corresponded to the development of flowering on the 7th to 9th day in *Arabidopsis*. To evaluate the potential functions of DNB members, GO assignments were used to classify the functions of DNB members during the critical transition of *Arabidopsis* flower development.

**Fig. 5:**
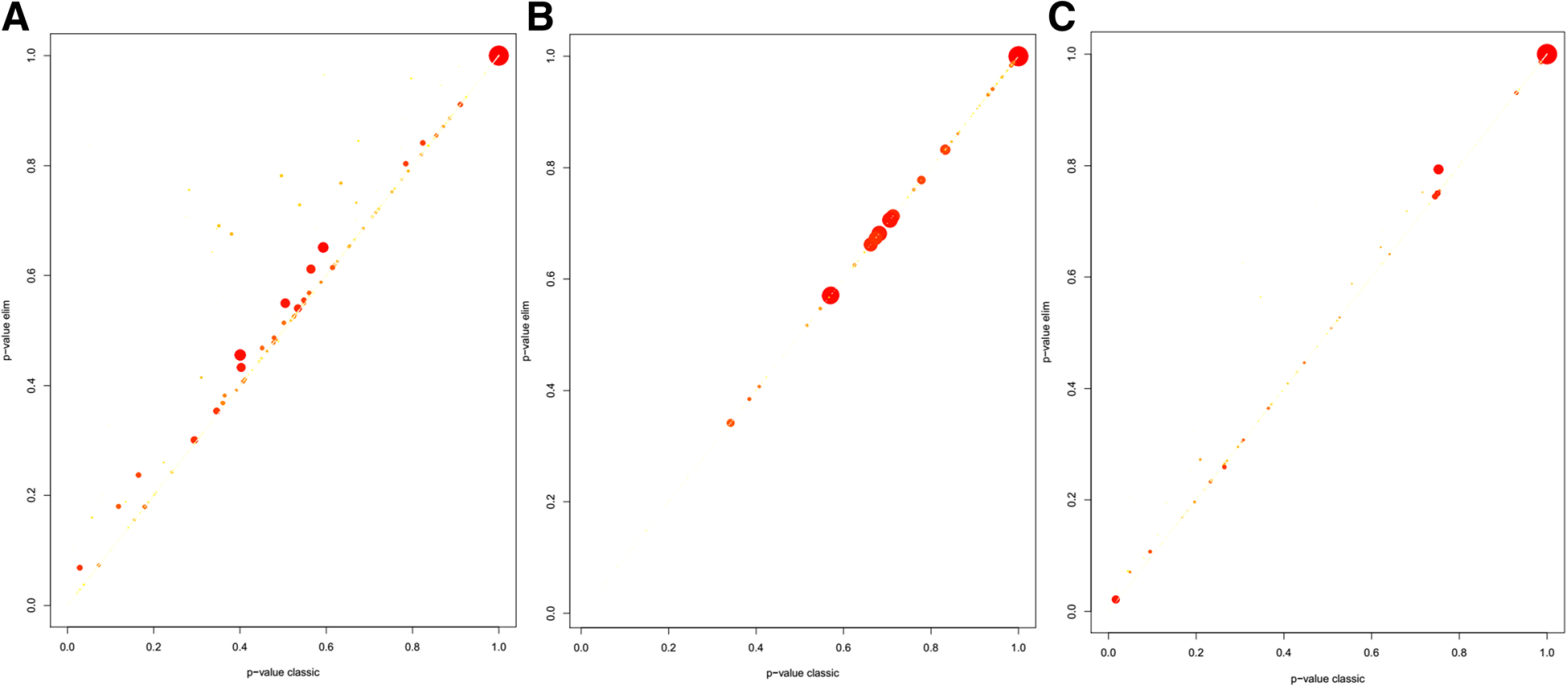
GO terms function enrichment analysis of DNB members. **a** biological process, (**b**) cellular component, (**c**) molecular function. The *p*-values are plotted on a linear scale. The size of the dot is proportional with the number of annotated genes for the respective GO term and its coloring represents the number of significantly differentially expressed genes, with the dark red points having more genes then the yellow ones

In the biological process category, two GO terms, i.e. small molecule catabolic process and plant-type cell wall biogenesis were enriched significantly in most DNB members (Fig. 5a). In the cellular component category, there were plenty of DNB members associated with AP-type membrane coat adaptor complex, secondary cell wall, and intrinsic component of mitochondrial inner membrane (Fig. 5b). In the molecular function category, ion antiporter activity was enriched significantly in most genes of the DNB module, and phosphate ion transmembrane transporter activity, serine-type endopeptidase inhibitor activity and L-ascorbic acid binding were similarly enriched in the DNB module (Fig. 5c).

**Fig. 6:**
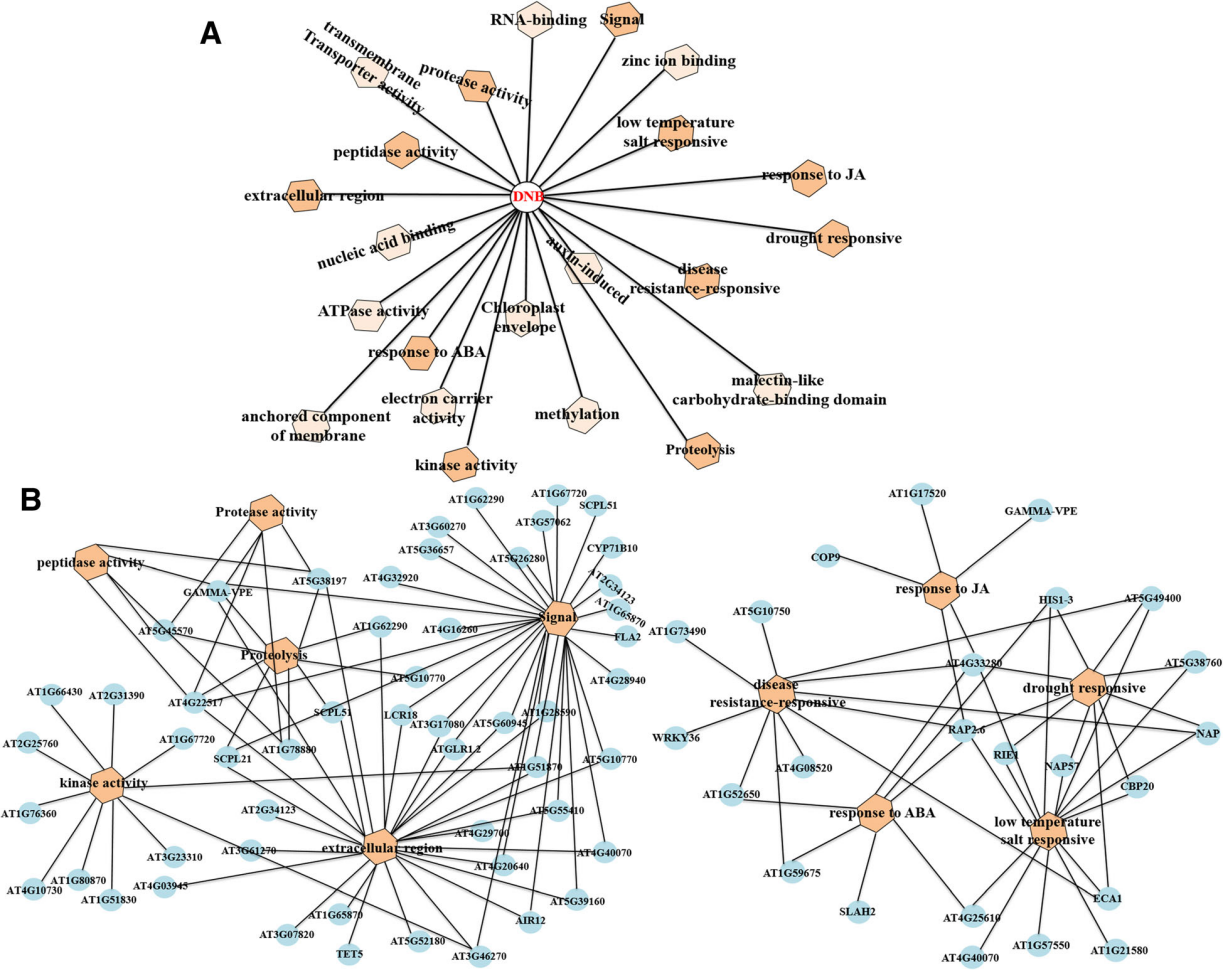
Transcriptional modules predicted to regulate the floral transition. **a** There are a number of GO terms (orange hexagons) which are significantly overrepresented (P, 0.01) in the DNB module. **b** Several GO terms (orange hexagons) that are significantly overrepresented (P, 0.01) within the DNB module (circles) are shown together with co-expressed genes (light blue Circles)

All the other overrepresented GO terms of DNB members in the three GO categories (biological process, cellular component, and molecular function) were listed in Table 2. And the metabolic pathways were listed in Additional file 9: Table S2. To further analyze key regulatory factors that control the phase transition from initiation to maturation in *Arabidopsis* flower development, we inferred a transcriptional module that links key regulatory factors with their potential targets (Fig. 6).

**Table 2:** Significance of GO terms obtained by R package topGO

GO	GO.ID	Term	Annotated	*P*-value
biological process	GO:0044282	small molecule catabolic process	196	0.0044
	GO:0009832	plant-type cell wall biogenesis	174	0.00428
	GO:0006643	membrane lipid metabolic process	101	0.00801
	GO:0009640	photomorphogenesis	95	0.00081
	GO:0032101	regulation of response to external stimu...	85	0.00713
	GO:0090691	formation of plant organ boundary	16	0.00943
	GO:0010244	response to low fluence blue light stimu...	9	0.00719
	GO:0010264	myo-inositol hexakisphosphate biosynthet...	8	0.00617
	GO:0000967	rRNA 5′-end processing	7	0.00401
	GO:0052746	inositol phosphorylation	5	0.00133
cellular component	GO:0030119	AP-type membrane coat adaptor complex	18	0.015
	GO:0031304	intrinsic component of mitochondrial inn...	18	0.052
	GO:0009531	secondary cell wall	16	0.053
	GO:0005880	nuclear microtubule	7	0.031
	GO:0016442	RISC complex	5	0.047
	GO:0031332	RNAi effector complex	5	0.047
	GO:0034098	VCP-NPL4-UFD1 AAA ATPase complex	3	0.014
	GO:0032541	cortical endoplasmic reticulum	2	0.022
	GO:0000312	plastid small ribosomal subunit	2	0.041
	GO:0005971	ribonucleoside-diphosphate reductase com...	2	0.051
molecular function	GO:0099516	ion antiporter activity	94	0.0125
	GO:0015114	phosphate ion transmembrane transporter ...	26	0.0019
	GO:0004867	serine-type endopeptidase inhibitor acti...	22	0.0018
	GO:0031418	L-ascorbic acid binding	13	0.008
	GO:0048038	quinone binding	10	0.0031
	GO:0005242	inward rectifier potassium channel activ...	10	0.013
	GO:0016639	oxidoreductase activity, acting on the C...	6	0.0041
	GO:0050284	sinapate 1-glucosyltransferase activity	4	0.004
	GO:0004148	dihydrolipoyl dehydrogenase activity	4	0.0069
	GO:0016629	12-oxophytodienoate reductase activity	3	0.0082

### The detected key genes of the critical transition

We not only discovered a critical transition which occurred between the seventh and ninth days of *Arabidopsis* flower development, but also found that 24 genes in this DNB module participate in stress reaction process, as well as in other floral-related pathways (Fig. 6b). Biological and abiotic stresses negatively affect plant growth and development including flowering and thus reduce productivity [49]. Adversity related genes included disease-, ABA-, JA-, drought-, low temperature and salt-related genes, i.e. CBP20, NAP, AT4G33280, RAP2.6, ECA1, AT5G38760, WRKY36, HIS1–3, AT1G73490, RIE1, AT1G52650, AT4G08520, AT1G59675, etc. Many of these genes such as RIE1, ECA1 and NAP were predominantly expressed from or after the 9th day which is the pollen formation stage. Thus, these genes might be play a vital role in the formation of microspores and the differentiation of pollen grains to a large extent.

The cap-binding protein complex (CBC) plays an important role in RNA metabolism because the CBC binds to the caps of all RNA polymerase II transcripts. As one subunit of the *Arabidopsis thaliana* CBC, the Cap Binding Protein 20 (CBP20) was determined to be involved in normal plant growth and development as well as RNA metabolism [50]. Studies have found that *Arabidopsis* cbp20 null mutant exhibited abnormal development of leaves and flowers and showed increased sensitivity to salt stress, which suggests that CBP20 has a synergistic effect in salt stress response [51]. Moreover, the drought-tolerant cbp20 mutant could maintain normal growth and development under drought stress, which might also point to a new cellular output mechanism as targets of the ABA regulatory pathway [52]. The plant specific NAM/ATAF1/2/CUC2 (NAC) transcription factors play important roles in abiotic stress-response signaling. We found that two NAC-like genes named NAP and NAP57 (activated by AP3/PI) as two of the important DNB members involved in a trifurcate feed-forward pathway of the drought stress response and their expression at the critical transition state was different from other periods.

Moreover, we also found NAP12 to be involved in the gametogenesis of *Arabidopsis* with two genes of ECA1 (Early Culture Abundant 1) gametogenesis-related family genes, AT5G60945 and AT5G36657. The ECA1 family proteins can be activated transcriptionally during the transition of microspores from the gametophytic to the embryogenic pathway. In addition, RIE1 and LEA (AT5G38760), two genes in the DNB module were found to be involved in the embryonic development of *Arabidopsis*. A RIE1 gene encoding a RING-H2 zinc-finger protein was identified in *Arabidopsis*, and it might be a membrane-associated protein, possibly relating to chloroplast. The late embryogenesis Abundant (LEA) protein family plays a role in drought stress tolerance. How to improve reproductive development by this transition depended on the interactions between regulatory factors. These regulators were important for signal transduction to control the transition of *Arabidopsis* flower development.

The APETALA2 (AP2) transcription factors (TFs) plays an important dynamic role in the embryo development, seedling built, flowering as well as stress response process [53, 54]. Two genes including RAP2.6 and AP2/B3-like (AT4G33280) were identified by DNB method. The *Arabidopsis* transcription factors RAP2.6 and AP2/ERF were found to be involved not only in ABA, salt and drought stress responses, but also in stamen emergence [55]. WRKY genes are a family of regulatory genes isolated from plants. WRKY proteins encoded by WRKY genes constitute a large family of plant-specific transcription factors. WRKY36, one of the WRKY gene family members, was identified in DNB module. Recent research found that UVR8 interacts with WRKY36 to regulate HY5 transcription and hypocotyl elongation in *Arabidopsis*, while we found that WRKY36 plays an important role in the critical transition [56].

As result of our analysis, 233 genes were identified to be highly fluctuated at the transition state and formed the DNB module. More importantly, a high percentage of genes with maximum expression in pollen was detected, which were predominantly expressed from or after the 9th day. For example, we found that two NAC-like genes named NAP and NAP57 (activated by AP3/PI) as two of the important DNB members were involved in a trifurcate feed-forward pathway of the drought stress response and they were up-regulated at the critical transition state [57]. Numerous studies have shown that class B MADS-box genes (AP3/PI) are crucial for stamen development [58]. Thus, the critical transition state detected during the formation of flowers was regarded as the pollen formation stage.

Some genes involved in cell differentiation were detected in this DNB module with predominant expression at late stages of flower development. These genes were predominantly expressed from or after the 9th day which is the pollen formation stage. For example, the regulator of ovule and seed development SEEDSTICK (STK) was significantly upregulated between days 7 and 9 [49]. DUO POLLEN1 (DUO1), a regulator of male germline development, was found for the first time to be expressed at the same stage [50]. And thus, these genes may be involved to a large extent in the differentiation of microspores into pollen grains. Gene Ontology (GO) analysis also showed that secondary cell wall synthesis related genes were enriched significantly in this critical transition (GO:0009531). The tapetum was developed from the secondary cell wall, which was the important component of pollen [59]. While many changes in gene expression may be owing to the activation of specific gene sets during the late pollen development, genes with significant regulatory functions such as genes coding APETALA2 (AP2) transcription factors and MADS-domain proteins often show intermittent expression at various stages of flower development [54, 55].

Genes involved in different plant hormone responses such as abscisic acid, auxin, and jasmonic acid were also detected as enriched significantly in this DNB module. This discovery is consistent with the known roles of these hormones in various biological processes in the late-stage flower development, such as the formation of stamen and pollen as well as the maturation of petals [60]. For example, AGL46 (AT2G28700), encoding the *Arabidopsis* MADS-box transcription factor was found to be involved in the CCKR signaling map, gonadotropin-releasing hormone receptor pathway, PDGF signaling pathway, interleukin signaling pathway, Ras Pathway and p38 MAPK pathway by KEGG analysis. Studies have shown that members of the MADS-box gene family play vital roles in flower development from early determination of floral meristem identity to later determination of floral organ primordial identity [58]. In a nutshell, the critical transition state detected during the formation of flowers and key regulators identified in the transition might answer the complex mechanisms of floral organ formation.

### A critical transition state of flower development in Rice

**Fig. 7:**
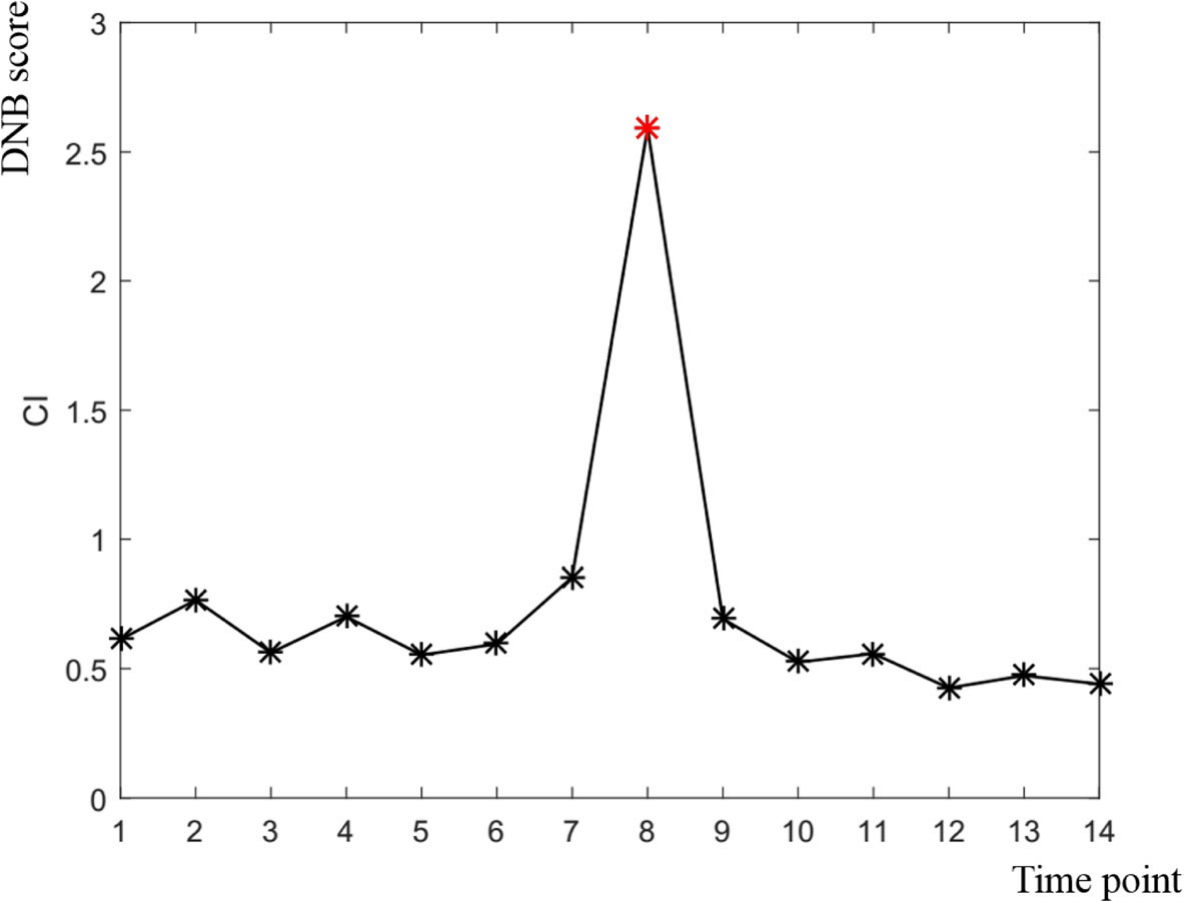
Identification of the DNB of flower development in rice. The DNB scores at 14 sampling time points is shown in the FIGURE. For the black curve, the DNB score increased sharply from the 7th point and reached the peak at the 8th point. The 8th sampling time point annotated by the red star is designated as a critical transition state, which corresponds to the development of specific floral organs (anther and pistil) in rice

To support the conclusions drawn, a similar analysis was performed on the flowering development data in rice. The dataset with spatio-temporal gene expression profiling throughout entire growth in rice was downloaded from the NCBI Gene Expression Omnibus (GEO) database under the accession number GSE21396 (www.ncbi.nlm.nih.gov/geo). The dataset include 15 time-points and 3 replicates for flowering development from initiation to maturity in rice [61]. To overcome the lack of distinction between the case sample and the control sample in this dataset, we compared the samples in both the previous and current time points. The samples in previous and current time points were designated as the control and case samples respectively. Thus, 15 different developmental stages from initial to mature were divided into 14 combinations for the detection of the critical transition. The pipeline of genome-wide dynamic network analysis was performed on the processed data with 14 combinations and a critical transition state of flower development was detected (Fig. 7). The critical transition state detected during the formation of flowers corresponded to the development of specific floral organs (anther and pistil) in rice [61].

Not only that, we also detected the DNB biomarkers with 206 genes that may herald the imminent critical transition during the formation of flowers. The genes detected in the critical transition state were listed in Additional file 10: Figure S3. The anther showed a unique growth characteristic in which most anther-specific genes were expressed only in a particular developmental stage [61]. We found that a pollen-specific gene (LOC4341399) coding for a pollen-specific leucine-rich repeat extensin-like protein was up-regulated in the critical transition. This result indicated that our researches can conduce to reveal the complex regulatory mechanisms of gene expression during anther growth and development as well as pollen germination.

The Gene Ontology (GO) analysis exhibited that protein kinase-encoding genes were significantly enriched among the down-regulated genes (Table 3). The results indicated that several signal transduction pathways involved in protein phosphorylation might undergo complex changes during this critical transition phase. Several transcription factors including 3 F-box-, 1 U-box-, 2 MYB-family genes were also up-regulated during this critical transition phase. Many studies have indicated that F-box proteins constituted one of the eukaryotic protein families, which played pivotal roles in regulating various developmental processes of plants. For example, a F-box gene DDF1 was a crucial genetic factor with pleiotropic effects in the development of rice floral organs [62]. CSA, a gene encoding the R2R3 MYB transcription factor which preferentially expressed in anther tapetal cells was identified [63]. These researches indicated that the F-box-, U-box-, MYB-family genes detected in this critical transition phase might be play pivotal roles in regulating anther development in rice. Other up- and down-regulated genes also play a crucial role in the process of rice pollen development.

**Table 3:** Functional categories for up- and down-regulated genes in the DNB module of rice flower development

Gene name	Fold change	Transition state	Description
Os04g0417400	6.80	up-regulated	U box domain containing protein.
LOC4340069	1.93	up-regulated	DNA-binding protein (Homeodomain-leucine zipper transcription factor)
LOC4346839	1.82	up-regulated	Disease resistance protein family protein.disease resistance protein RGA5-like
LOC4341399	1.74	up-regulated	pollen-specific leucine-rich repeat extensin-like protein 1
LOC4326659	1.59	up-regulated	Cytochrome P450 monooxygenase CYP72A5
LOC4338660	1.56	up-regulated	UDP-sulfoquinovose synthase, chloroplast precursor (Sulfolipid biosynthesis protein)
LOC4345661	1.43	up-regulated	Myb-like protein (transcription factor CSA-like)
LOC4324767	1.36	up-regulated	TPR-like domain containing protein.
LOC4339280	1.36	up-regulated	Cyclin-like F-box domain containing protein (F-box protein At5g46170)
LOC4332901	1.32	up-regulated	Sugar transporter-like protein
LOC4324342	1.32	up-regulated	Plant lipid transfer/seed storage/trypsin-alpha amylase inhibitor domain containing protein
LOC4351030	1.32	up-regulated	Hypothetical protein
LOC4341957	1.31	up-regulated	Macrophage migration inhibitory factor family protein
LOC112936214	1.32	up-regulated	eukaryotic cysteine peptidase active site family protein (putative F-box protein At5g15660)
LOC4324302	1.37	up-regulated	Myb-related protein 5 (transcription factor MYB59)
LOC4338652	1.31	up-regulated	ABC transporter G family member 23
LOC4338416	0.79	down-regulated	ABC-1 domain containing protein (protein ACTIVITY OF BC1 COMPLEX KINASE 3, chloroplastic)
LOC4329899	0.71	down-regulated	Galactosyltransferase associated protein kinase p58/GTA (Cell division cycle 2-like 2)
LOC4348579	0.78	down-regulated	Protein kinase APK1B (EC 2.7.1.-)
LOC4338491	0.76	down-regulated	Ribulose bisphosphate carboxylase
LOC4350151	0.76	down-regulated	probable sucrose-phosphate synthase 5
LOC4349274	0.75	down-regulated	Zn-finger, RING domain containing protein (NEP1-interacting protein 1)
LOC4335569	0.69	down-regulated	Helicase-like protein
LOC4324503	0.57	down-regulated	Ribosomal protein S8 family protein.
LOC4335392	0.49	down-regulated	Allergen V5/Tpx-1 related family protein (pathogenesis-related protein 1)
LOC9272032	0.35	down-regulated	probable LRR receptor-like serine/ threonine-protein kinase At1g51810

These results supported the conclusions drawn on the development of *Arabidopsis* flower. Therefore, the transcriptomic profiling analysis using DNB and MARROMI method could provide new insight to characterize the formation of flowers and detect key regulatory factors that might control the transition from initiation to maturation in Arabidopsis flower development.

## Discussion

The formation of flowers is one of the main models for studying the regulation mechanisms of plant growth and development. In past botanical studies, the floral transition was recognized as the progression from vegetative growth to reproductive growth [34, 38]. Although some network-based bioinformatics analyses attempted to identify the phase transition which indicate the progression from vegetative growth to reproductive, there is no research on identifying the critical transition stage of flower formation from the time of initiation to maturation. Differentiation of floral organs is more complex than other parts of the plant, especially the formation of pollen [61]. In this study, we discovered a critical transition stage of *Arabidopsis* flower development using the DNB theory. It is the first time that DNB model has been used in plant research. We found that the phase transition occurred between the seventh and ninth days of *Arabidopsis* flower development. The development of flowers on a given inflorescence was uniformly synchronized until day 7, then the expression of flowering related genes has changed dramatically. Not only that, we found that this critical transition state detected during the formation of flowers corresponded to the development of specific floral organs (anther). We also detected the DNB members composed by 233 genes that may herald the imminent critical transition during the formation of flowers. Moreover, the interactions between these genes also regulate the critical transition process.

To construct the gene regulatory network controlling the flowering transition, we applied NARROMI algorithm which can reduce the noisy, redundant and indirect regulations on the expression data of the transition-specific genes. We further found that 24 genes in this DNB module participate in stress reaction process, as well as in other floral-related pathways such as gametogenesis and embryo development. Our research here suggests that previously unknown regulatory genes identified in the transition region might be through known regulatory mechanisms to promote the formation of flowers. Therefore, a further study of co-expressed genes in the transition region might answer the connection between co-expressed genes and critical transition.

In addition, the highlight of this article is the effective combination of DNB and NARROMI methods. In contrast to the traditional methods or biomarkers based on differential expressions of molecules, DNB method can identify a critical transition state of a complex biological process based on collective fluctuations and correlations of different metabolic molecules at the network level. Thus, even if there are no significant differential expressions in the before-transition state and the critical transition state, we can detect significant differential correlations and deviations in these two states by DNB method [35].

In contrast to the traditional methods to construct the gene regulatory network controlling the flowering transition, NARROMI algorithm can reduce the noisy, redundant and indirect regulations on the expression data of the transition-specific genes. Firstly, NARROMI algorithm can calculate the causal intensity between gene pairs by quantifying the non-linear correlation mutual information (MI), so that we can reduce interference regulators with low correlation. Then, ordinary differential equation-based recursive optimization (RO) is used to gradually reduce the redundant and indirect regulations. Ultimately, we obtained a topology of a non-linear sparse gene regulatory network by network integration which was the most similar gene regulatory network to the real network. NARROMI algorithm could be regard as a further improvement of the DNB model.

We also performed a similar analysis of temporal gene expression profiling dataset of rice flower development to support the conclusions drawn. Ultimately, we detected a critical transition state of flower development in rice. This critical transition state detected during the formation of flowers corresponded to the development of specific floral organs (anther and pistil) in rice. Not only that, we also detected the DNB members composed by 206 genes that may herald the imminent critical transition during the formation of flowers. We found that the genes detected in this critical transition phase might be play pivotal roles in regulating anther development in rice. These results supported the conclusions drawn on the development of Arabidopsis flowering. In addition to the application of DNB and NARROMI algorithm to detect the critical state of Arabidopsis flowering transition, they can be used to detect the critical transition of any biological process.

## Conclusions

We studied the flower developmental phase transition from the time of initiation to maturation in *Arabidopsis* using dynamic network biomarker model. The critical transition state of flowering development was detected and a cluster of genes as dynamic network biomarkers controlling the phase transition of flower development from the initiation to maturation were identified. In contrast to the traditional methods based on differential gene expression analysis, our analysis can exploit collective fluctuations and correlations of different metabolites at a network level to identify a critical transition state of a complex biological process. We also detected the dynamic network biomarkers composed of several genes that may herald the imminent critical transition during the formation of flowers. To construct the gene regulatory network controlling the flowering transition, we applied NARROMI algorithm on the expression data of the transition-specific genes. The redundancy reduction technique-based network reconstruction method NARROMI algorithm could be regard as a further improvement of the dynamic network biomarker model, which can reduce noisy and indirect regulations to improve the accuracy of the network inference. Our research suggests that the critical transition state detected during the formation of flowers and key regulators identified in the transition might answer the complex mechanisms of floral organ formation. The bioinformatics analysis used in this work can also be used to detect the critical state of any biological process.

## Methods

### Gene expression data collection

The *Arabidopsis* flower developmental gene expression profiling dataset was downloaded from the NCBI Gene Expression Omnibus (GEO) database under the accession number GSE64581 (www.ncbi.nlm.nih.gov/geo). The dataset includes 14 different time-points with 3 replicates of gene expression data from initiation to maturity, i.e. 0d, 1d, 1.5d, 2d, 2.5d, 3d, 3.5d, 4d, 4.5d, 5d, 7d, 9d, 11d and 13d (Fig. 6a). The data was downloaded from GEO database already pre-processed, and we followed procedure outlined in Ryan et al., 2015 and some other protocol. Ryan et al.’s article is an Open Access article distributed under the terms of the Creative Commons Attribution License (http://creativecommons.org/licenses/by/4.0).

The data was downloaded from GEO database was a time-courses gene expression matrix. The horizontal row indicated the expression value of one gene in different samples, and the column represented the expression value of the gene pool of one sample. We used the function ‘genelowvalfilter’ in MATLAB to filter genes. After removing those genes whose expression values were less than a certain threshold, we used the remaining genes for further processing. Because some of the transcriptional changes are caused by specific gene regulatory events or tremendous alterations in floral size and morphology during the flower development, we compared the gene expression level between consecutive as well as neighbor (within a 2d time interval) time-points to minimize the effect of morphological changes [8].

### Network inference

In general, a mathematical model based on mass action kinetics and Michaelis–Menten kinetics can describe the transcriptional regulation process [64]. However, the noise inherited in the gene expression data can decrease the performance of these models [65]. Therefore, to improve the accuracy of the network inference, NARROMI algorithm was used to reduce noisy, redundant and indirect regulations [29]. It initially calculates the causal strength between gene pairs by quantifying the non-linear correlation mutual information (MI), which can reduce noisy regulations in gene expression data [66]. Then, ordinary differential equation-based recursive optimization (RO) is used to gradually reduce the redundant and indirect regulations to obtain a final topology of a non-linear sparse gene regulatory network (Fig. 6c). The script that was used to apply the NARROMI algorithm was listed in Additional file 11: Script 1. By comparing with GENIE3, ARACNE etc., NARROMI outperformed these popular methods in most cases, thereby verifying its effectiveness [29].

To obtain a more reliable gene co-expression network, a composite index is proposed combining mutual information(MI) and recursive optimization(RO)-based estimation of parameters:

$$ \beta =\operatorname{sign}\left({\beta}^{\mathrm{RO}}\right)\left(\omega \left|{\beta}^{\mathrm{RO}}\right|+\left(1-\omega \right){\beta}^{\mathrm{MI}}\right) $$

where *β*^MI^ is the MI value which is positive, *β*^RO^ is the regulatory strength (positive or negative) inferred by RO algorithm, sign(*β*^RO^) is the sign (+) of *β*^RO^, |*β*^RO^| is the absolute of *β*^RO^ and parameter ω is the weighting coefficient. The final regulatory strength is decided by the parameter *β*, and the network topology is then determined.

### GO enrichment and visualization

The identified DNB members were analyzed for functional enrichment analysis by R package topGO 2.30.1. The topGO package provided tools for testing GO terms while accounting for the topology of the GO graph. Different test statistics and different methods for eliminating local similarities and dependencies between GO terms can be implemented and applied. The enrichment analysis process consists of input of normalized gene expression measurements, gene-wise correlation or differential expression analysis, enrichment analysis of GO terms, interpretation and visualization of the results. The data preparation process is critical before running the enrichment test. The user needs to provide the gene universe, GO annotations and either a criteria for selecting interesting genes (e.g. differentially expressed genes) from the gene universe or a score associated with each gene. The GO database used in our research was a set of annotation maps describing the entire Gene Ontology assembled using data from GO Version: 3.5.0.

Moreover, we also analyzed key regulatory factors and key metabolic pathways that were closely related to DNB of *Arabidopsis* flower development from initiation to maturation time (Fig. 6d). The results of the gene regulatory networks were imported in Cytoscape (www.cytoscape.org) for visualization.

### Regulatory circuits and regulators prediction

We further predicted the regulatory circuits and regulators controlling the critical transition based on the *Arabidopsis* Gene Regulatory Information Server (AGRIS; http://arabidopsis.med.ohio-state.edu) [67]. We can obtain a comprehensive resource for *Arabidopsis* gene regulatory studies from the AGRIS. There are three interlinked databases, AtTFDB, AtcisDB and AtRegNet. The updated and comprehensive information on transcription factors (TFs) could be obtain from AtTFDB, which is the key to study the *Arabidopsis* gene regulatory networks.

## Additional files

Additional file 1: **Figure S4.** Comparison results of the DNB-based method, bootstrap analysis. (DOCX 313 kb)

Additional file 2: **Figure S1.** List of genes for the DNB module during *Arabidopsis* flower development. (TXT 2 kb)

Additional file 3: **Figure S2.** Detailed description of the identified DNB module for *Arabidopsis* flower development. (TXT 95 kb)

Additional file 4: **Table S1.** Functional categories for the DNB members of Arabidopsis flower development. (TXT 7 kb)

Additional file 5: **Table S3.** Network inference file. (TXT 32 kb)

Additional file 6: **Table S4.** The degree of correlation between each pair of DNB genes during Arabidopsis flower development. (TXT 7 kb)

Additional file 7: **Table S5.** Node properties file for the network. (TXT 4 kb)

Additional file 8: **Table S6.** The gene regulatory network Cytoscape file. (TXT 7 kb)

Additional file 9: **Table S2.** The DNB members of Arabidopsis flower development enriched GO terms and metabolic pathways. (TXT 2 kb)

Additional file 10: **Figure S3.** The DNB members of flower development in rice. (TXT 9 kb)

Additional file 11: **Script 1.** Tair_DNB_narromi. (DOCX 14 kb)

## Abbreviations

ABA: Abscisic acid
CBC: Cap-binding protein complex
DNB: Dynamic network biomarker
ECA1: Early Culture Abundant 1
GRN: Gene regulatory network
LEA: Late embryogenesis Abundant
MI: Mutual information
PCC: Pearson correlation coefficient
RO: Recursive optimization
