# Supplementary material for: Genome-wide dynamic network analysis reveals a critical transition state of flower development in Arabidopsis
Source: BMC Plant Biol. 2019 Jan 7;19:11. doi: 10.1186/s12870-018-1589-6 (PMC6323737; doi:10.1186/s12870-018-1589-6)
Supplement: Supplementary file 1 — Figure S4. Comparison results of the DNB-based method, bootstrap analysis. (DOCX 313 kb) [file 12870_2018_1589_MOESM1_ESM.docx]

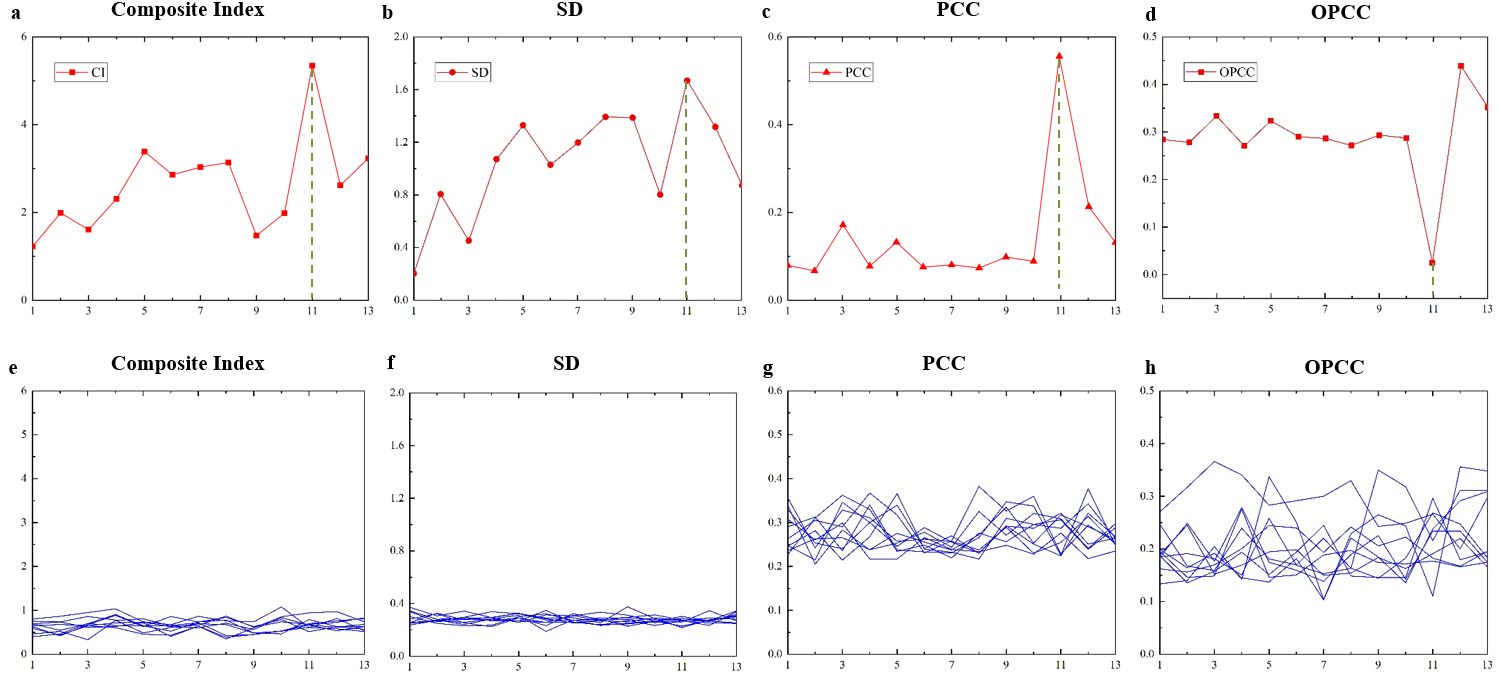


Figure S4 Comparison results of the DNB-based method, bootstrap analysis. In order to show the biological significance, we compared our method with the existing methods for flowering development in *Arabidopsis*. (a) Mean standard deviation (SD) for the case group data of the identified DNB. (b) Mean PCC (PCC in the DNB) for the case group data of the identified DNB. (c) Mean OPCC (PCC between the DNB and other molecules) for the case group data of the identified DNB. (d) Composite index for the case group data of the identified DNB. (e) Mean standard deviation (SD) for the 10 sets of randomly selected genes (bootstrap analysis); each set of them has the same number of members as the identified DNB group. (f) Mean PCC for the 10 sets of randomly selected genes (bootstrap analysis). (g) Mean OPCC for the 10 sets of randomly selected genes (bootstrap analysis). (h) Composite indices for the 10 sets of randomly selected genes (bootstrap analysis). The dotted green line indicates the pre-disease period.
