# Supplementary material for: Genome-wide dynamic network analysis reveals a critical transition state of flower development in Arabidopsis
Source: BMC Plant Biol. 2019 Jan 7;19:11. doi: 10.1186/s12870-018-1589-6 (PMC6323737; doi:10.1186/s12870-018-1589-6)
Supplement: Supplementary file 11 — Script 1. Tair_DNB_narromi. (DOCX 14 kb) [file 12870_2018_1589_MOESM11_ESM.docx]

load tair_DNB_data.mat;

lamda = 1;

alpha = 0.05; % parameter for MI correlation.

beta = 0.05; % parameter for RO.

t = 0.6; % parameter for the rate of RO in the integration.

% As an example, we choose part of the expression.

TF_list = tair1TGlist(:,:);

TF_expression = tair1TGexpression(:,:);

network = zeros(size(TF_list,1),size(TF_list,1));

network_v = network;

netsig = network;

%%

for i=1:size(TF_list,1)

y = TF_expression(i,:);

X = [TF_expression(1:i-1,:);TF_expression(i+1:size(TF_expression,1),:)];

[net,net_value,sig]=narromi(y',X',lamda,alpha, beta, t) ;

network(i,1:i-1) = net(1:i-1);network(i,i+1:size(TF_expression,1)) = net(i:end);

network_v(i,1:i-1) = net_value(1:i-1);

network_v(i,i+1:size(TF_expression,1)) = net(i:end);

netsig(i,1:i-1) = sig(1:i-1); netsig(i,i+1:size(TF_expression,1)) = sig(i:end);

i

end

% Output the network

significance = 0.00001;

network_sig = zeros(size(netsig)) ;

network_sig(find(netsig<=significance)) = 1 ;

network_sig(logical(eye(size(network_sig)))) = 0;

[testfile_network]=Connect_for_cytoscape_pvalue(network_sig',network_v',netsig',TF_list,TF_list) ;

network_size=size(testfile_network,1);

fprintf('NOTICE:\nThe Size of the Inferred Network is %d.\n',network_size);

a=testfile_network';

fid=fopen('network_inferred.txt','w');

fprintf(fid,'%s %s %.6f %.3e\n',a{:}) ;

fclose(fid);

fprintf('NOTICE:\nPlease Find the Network File in the Matlab Current Folder.\n')
